# Supplementary material for: Histamine synthesis and transport are coupled in axon terminals via a dual quality control system
Source: EMBO J. 2024 Sep 6;43(20):4. doi: 10.1038/s44318-024-00223-0 (PMC11480334; doi:10.1038/s44318-024-00223-0)
Supplement: Supplementary file 2 — Table EV1 [file 44318_2024_223_MOESM2_ESM.docx]

Table EV1. Hdc binding protein candidates.

| **Gene** | **#CG** | **Description** | **Location** | **Mascot score** |
| --- | --- | --- | --- | --- |
| *NSF1* | *CG1618* | Disassembly SNARE complexes following synaptic vesicles fusion | Synaptic vesicles | 791 |
| *Tm1* | *CG4898* | Muscle contraction | Cytosol | 494 |
| *CG7920* | *CG7920* | Acetate CoA-transferase | Cytosol and mitochondria | 125 |
| *Hsp83* | *CG1242* | Molecular chaperone | Cytosol | 64 |
