# Supplementary material for: Histamine synthesis and transport are coupled in axon terminals via a dual quality control system
Source: EMBO J. 2024 Sep 6;43(20):4. doi: 10.1038/s44318-024-00223-0 (PMC11480334; doi:10.1038/s44318-024-00223-0)
Supplement: Supplementary file 3 — Table EV2 [file 44318_2024_223_MOESM3_ESM.docx]

Table EV2. E3 ligases screen. The levels of Hdc-mCherry were assessed via stereo microscope, with "YES" indicating increased mCherry signals. No instances of reduced Hdc-mCherry signals were observed. Tissue expression data were sourced from the "Expression Data" section in FlyBase.

| **#CG** | **Lab stock** | **Tsinghua stock** | **Bloomington**  **stock** | **VDRC stock** | **expression in eye** | **Results** |
| --- | --- | --- | --- | --- | --- | --- |
|  |  |  |  |  |  |  |
| CG2679 | B33 |  |  |  | Adult Male Eye | NO |
| CG10277 | R195 |  |  |  | moderate | NO |
|  |  |  |  |  |  |  |
|  |  |  |  |  |  |  |
| cg3356 |  |  |  | v101229 v34599 | moderate | NO |
| cg4238 |  |  |  |  | no |  |
| cg5087 |  |  |  |  | moderate | NO |
| cg42797 | B159 |  |  |  | low | NO |
| CG42574 | A249 |  |  |  | moderate | NO |
| CG11734 | B164 |  |  |  | moderate | NO |
| CG9153 | A317 |  |  |  | moderate | NO |
| CG8184 | A5 |  |  |  | moderate | NO |
| CG9484 | H384 |  |  |  |  | NO |
| CG42279 | L97 |  |  |  | moderately high | NO |
| CG4943 | M367 |  |  |  | moderate | NO |
| CG4244 |  |  |  |  | moderate | NO |
| CG6190 | B95 |  |  |  | moderate | NO |
| CG5604 |  |  |  |  | moderate | NO |
|  |  |  |  |  |  |  |
|  |  |  |  |  |  |  |
| CG8293 | I115 |  |  |  | high | NO |
| CG12284 | H219 |  |  |  | moderate | NO |
|  |  |  |  |  |  |  |
|  |  |  |  |  |  |  |
| cg3526 |  | TH04130.N |  |  | no data | NO |
| cg15286 |  | TH03952.N |  |  | testis specific | NO |
| cg31642 |  | TH04211.N |  |  | testis specific | NO |
| cg31835 | K258 |  |  |  | no data | NO |
| cg42585 | K258 |  |  |  | no data | NO |
| CG11984 | J169 |  |  |  | moderate | NO |
|  |  |  |  |  |  |  |
|  |  |  |  |  |  |  |
| cg3894 | M88 |  |  |  | moderate | NO |
| cg7326 | G245 |  |  |  | low | NO |
| cg13994 |  |  |  | v31794 v48622 | moderate | NO |
| cg14646 |  |  |  | v103770 | moderate | NO |
| cg42707 |  |  |  | v108194 v45386 | testis specific | NO |
| cg46284 |  |  | 41637 44094 |  | low | NO |
| cg14472 | J191 |  |  |  | moderate | YES |
|  |  |  |  |  |  |  |
|  |  |  |  |  |  |  |
| cg10542 |  | THU1934 |  |  | low | NO |
| CG7037 | Q136 |  |  |  | low | NO |
| cg1317 |  |  |  | v4106 | high | NO |
| cg2617 | J194 |  |  |  | low | NO |
| cg2681 |  |  |  | v110791 v33549 | high | NO |
| cg2991 |  |  |  | v2604 v2605 | high | NO |
| cg4080 |  |  |  |  | moderate | NO |
| cg4325 |  |  |  |  | no |  |
| cg4813 | K169 |  |  |  | low | NO |
| cg5334 |  |  |  |  | no |  |
| cg5347 |  |  |  |  | no |  |
| cg5555 | S6 |  |  |  | moderate | NO |
| cg6923 | H68 |  |  |  | low | NO |
| cg7376 |  |  |  |  | no |  |
| cg7694 |  |  |  |  | low | NO |
| CG8141 |  |  |  |  | no |  |
| cg8910 | U16 |  |  |  | low | NO |
| cg9014 |  |  |  |  | no |  |
| cg9855 |  |  |  |  |  |  |
| cg9941 | A49 |  |  |  | moderate | NO |
| cg10761 |  |  |  |  |  |  |
| cg10916 |  |  |  |  |  |  |
| cg11360 | C274 |  |  |  | low | NO |
| cg11414 | K206 |  |  |  | moderate | NO |
| cg12099 |  |  |  |  |  |  |
| cg12477 |  |  |  |  |  |  |
| cg13344 | M35 |  |  |  | low | NO |
| cg13442 |  | THU1727 |  |  | no | NO |
| cg13481 |  |  |  |  |  |  |
| cg13605 |  |  |  |  |  |  |
| cg14435 |  |  |  |  |  |  |
| cg14983 |  |  |  |  |  |  |
| cg15141 | E150 |  |  |  | moderate | NO |
| cg15814 |  |  |  |  |  |  |
| cg16781 |  |  |  |  |  |  |
| cg17019 | K227 |  |  |  | moderate | NO |
| cg17329 |  |  |  |  |  |  |
| cg17717 |  |  |  |  |  |  |
| cg17991 |  |  |  |  |  |  |
| cg31807 |  |  |  |  |  |  |
| cg32847 |  |  | 65067 |  | no | NO |
| cg33552 |  |  |  |  |  |  |
| cg34308 |  |  |  |  |  |  |
| cg34375 |  |  |  |  |  |  |
| CG31716 | M244 |  |  |  | moderate | NO |
| CG10981 |  | THU2593 |  |  | moderate | NO |
| CG12489 |  |  |  |  |  |  |
| CG3039 |  |  |  |  |  |  |
| CG3929 | A95 |  |  |  | low | NO |
| CG17033 |  |  |  |  |  |  |
| CG12812 |  | THU1165 |  |  | no | NO |
| CG10263 | E151 |  |  |  | low | NO |
| CG32592 | Q66 |  |  |  | low | NO |
| CG11982 |  |  |  |  |  |  |
| CG31357 |  |  |  |  |  |  |
| CG6752 | J24 |  |  |  | no | NO |
| cg34440 | L313 |  |  |  | no | NO |
| CG32210 |  |  |  |  |  |  |
| CG4973 | I112 |  |  |  | low | NO |
| CG5841 |  |  |  |  |  |  |
| CG17492 |  |  |  |  |  |  |
| CG7184 | M206 |  |  |  | low | NO |
| CG3241 | G89 |  |  |  | low | NO |
| CG1134 |  |  |  |  |  |  |
| CG9381 | G19 |  |  |  | modearte | NO |
| CG13025 | K240 |  |  |  | no | NO |
| CG11988 | G97 |  |  |  | low | NO |
| CG5140 |  |  |  |  |  |  |
| CG11329 |  |  |  |  |  |  |
| CG7081 | B60 |  |  |  | moderate | NO |
| CG7864 | B58 |  |  |  | low | NO |
| CG3639 |  | TH01879.N |  |  | low | NO |
| CG5212 |  |  |  |  |  |  |
| CG4909 | F127 |  |  |  | low | NO |
| CG43726 | M22 |  |  |  | no | NO |
| CG16947 | R77 |  |  |  | low | NO |
| CG32850 | I266 |  |  |  | high | NO |
| CG8786 | L331 |  |  |  | moderate | NO |
| CG16807 |  |  |  |  |  |  |
| CG5595 | G116 |  |  |  | low | NO |
| CG9949 | L311 |  |  |  | moderate | NO |
| CG13030 |  |  |  |  |  |  |
| CG1937 |  | TH01506.N |  |  | moderate | NO |
| CG3231 | A101 |  |  |  | low | NO |
| CG11281 |  |  |  |  |  |  |
| CG8974 |  |  |  | XJW |  | NO |
| CG32581 |  |  |  | XJW |  | NO |
| CG3295 | K156 |  |  |  |  | NO |
| CG3647 |  | THU5569 |  |  | moderate | NO |
| CG8780 |  |  |  |  |  |  |
| CG15104 | I4 |  |  |  | low | NO |
| CG10961 |  |  |  |  |  |  |
| CG2304 | C124 |  |  |  | moderate | NO |
| CG9086 |  |  | 31374 |  | no | NO |
| CG42593 |  |  |  |  |  |  |
| CG32350 |  |  |  |  |  |  |
|  |  |  |  |  |  |  |
|  |  |  |  |  |  |  |
| CG5659 | M245 |  |  |  | low | NO |
| CG5709 | A190 |  |  |  | moderate | NO |
| cg12362 |  |  |  |  |  |  |
| cg33144 | J53 |  |  |  | moderate | NO |
| CG11321 |  |  |  |  |  |  |
| CG10523 | L113 |  |  |  | low | NO |
|  |  |  |  |  |  |  |
|  |  |  |  |  |  |  |
| CG16982 | H24 |  |  |  | moderate | NO |
| CG16988 |  | TH04129.N |  |  | no | NO |
| CG8998 |  |  |  |  |  |  |
|  |  |  |  |  |  |  |
|  |  |  |  |  |  |  |
| CG5206 | N93 |  |  |  | moderate | NO |
| cg8419 |  |  |  |  |  |  |
| CG12218 | E190 |  |  |  | moderate | NO |
| CG15105 | E328 |  |  |  | no | NO |
| CG31721 | U17 |  |  |  | no | NO |
|  |  |  |  |  |  |  |
|  |  |  |  |  |  |  |
| cg2218 | S50 |  |  |  | moderate | NO |
| cg6179 | R17 |  |  |  | low | NO |
| cg7747 | C33 |  |  |  | low | NO |
| CG5519 | H158 |  |  |  | low | NO |
| CG5203 | H286 |  |  |  | moderate | NO |
| CG11070 |  |  |  |  |  |  |
| CG9934 | E330 |  |  |  | low | NO |
|  |  |  |  |  |  |  |
|  |  |  |  |  |  |  |
| CG8272 |  |  | 31361 |  | low | NO |
| CG9003 | K250 |  |  |  | low | NO |
| CG12402 | J1 |  |  |  | no | NO |
| CG13766 | K123 |  |  |  | no | NO |
| CG32085 | S125 |  |  |  | low | NO |
| CG13213 | A155 |  |  |  | moderate | NO |
| CG1839 |  |  | 31363 |  | high | NO |
| CG4221 |  |  | 31065 60461 |  | low | NO |
| CG2010 | S44 |  |  |  | no | NO |
| CG11033 | H129 |  |  |  | moderate | NO |
| CG9952 |  |  | 31357 |  | moderate | NO |
| CG9772 |  | THU0653 |  |  | no | NO |
| CG15010 | G206 |  |  |  | moderate | NO |
| CG9144 | A103 |  |  |  | low | NO |
| CG3412 | J225 |  |  |  | oderate | NO |
| CG2247 |  |  | 31486 |  | low | NO |
| CG4911 | C139 |  |  |  | no | NO |
| CG5003 | B172 |  |  |  | low | NO |
| CG5961 | R224 |  |  |  | low | NO |
| CG7148 | S90 |  |  |  | low | NO |
| CG7707 |  |  | 31367 |  | no | NO |
| CG9316 |  |  | 31485 |  | no | NO |
| CG11044 |  |  | 31364 |  | no | NO |
| CG11658 | N45 |  |  |  | low | NO |
| CG12520 | J19 |  |  |  | no | NO |
| CG13085 | K113 |  |  |  | low | NO |
| CG13088 |  |  |  |  |  |  |
| CG14317 |  |  |  |  |  |  |
| CG14937 |  |  | 31483 |  | no | NO |
| CG30466 |  |  | 31372 |  | low | NO |
| CG31633 |  |  |  |  |  |  |
| CG32221 | S136 |  |  |  | moderate | NO |
| CG34025 |  |  |  |  |  |  |
| CG11866 |  |  | 31064 |  | moderate | NO |
| CG9461 |  | THU4006 |  |  | low | NO |
| CG6758 |  |  | 31366 |  | low | NO |
| CG12765 |  |  | 31060 |  | low | NO |
| CG4643 |  |  | 31096 |  | moderate | NO |
| CG8873 | U81 |  |  |  | low | NO |
| CG15437 |  |  | 31059 |  | moderate | NO |
| CG10855 |  |  | 31487 |  | low | NO |
| CG3428 |  |  |  |  |  |  |
| CG10800 | B169 |  |  |  | low | NO |
