# Supplementary figures and images for: Histamine synthesis and transport are coupled in axon terminals via a dual quality control system
Source: EMBO J. 2024 Sep 6;43(20):4. doi: 10.1038/s44318-024-00223-0 (PMC11480334; doi:10.1038/s44318-024-00223-0)

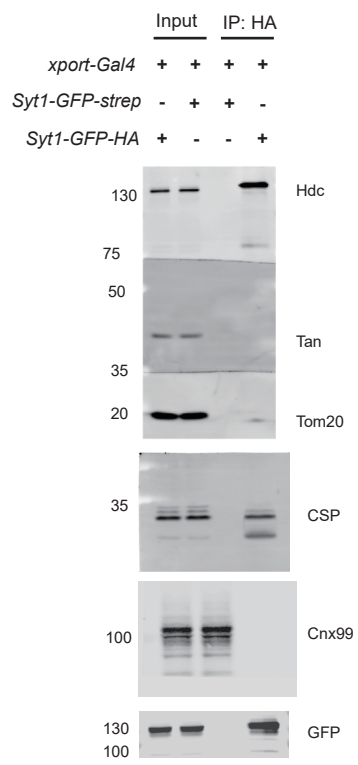

Supplement: Supplementary file 5 — Source data Fig. 2 [file 44318_2024_223_MOESM5_ESM.zip › figure 2/A/purify.pdf]

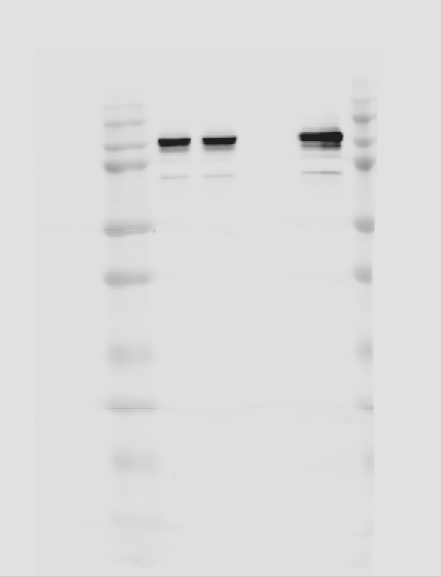

Supplement: Supplementary file 5 — Source data Fig. 2 [file 44318_2024_223_MOESM5_ESM.zip › figure 2/A/IP HA.tif]

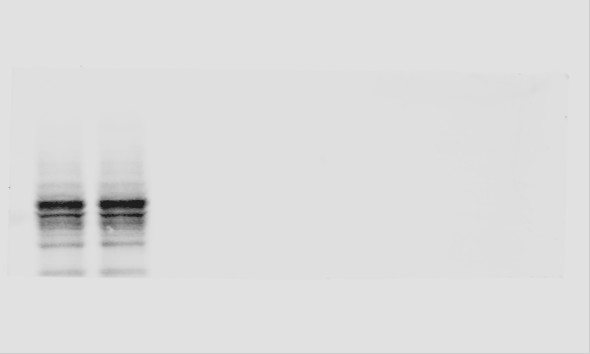

Supplement: Supplementary file 5 — Source data Fig. 2 [file 44318_2024_223_MOESM5_ESM.zip › figure 2/A/ER.tif]

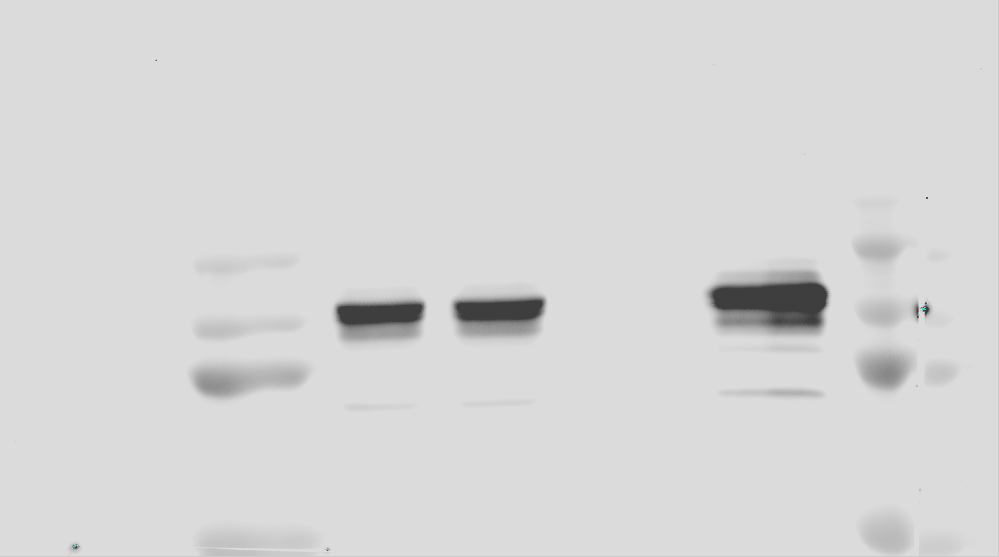

Supplement: Supplementary file 5 — Source data Fig. 2 [file 44318_2024_223_MOESM5_ESM.zip › figure 2/A/IP.tif]

|                |   |   |   |
|----------------|---|---|---|
| Hdc-mCherry    | + | - | - |
| Hdc-N-mCherry  | - | + | - |
| Hdc-dN-mCherry | - | - | + |
| NSF1-myc       | + | + | + |

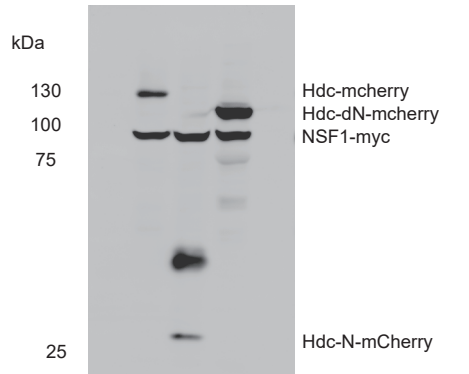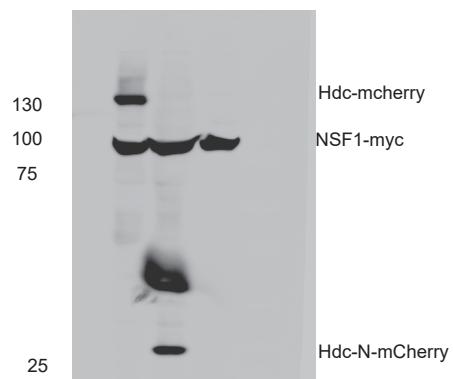

Supplement: Supplementary file 6 — Source data Fig. 3 [file 44318_2024_223_MOESM6_ESM.zip › figure 3/C/coIP.pdf]

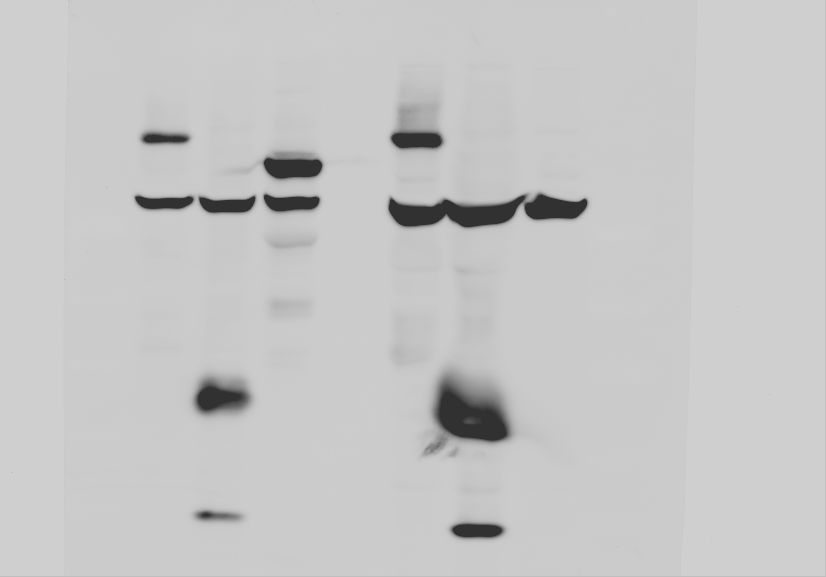

Supplement: Supplementary file 6 — Source data Fig. 3 [file 44318_2024_223_MOESM6_ESM.zip › figure 3/C/coIP.tif]

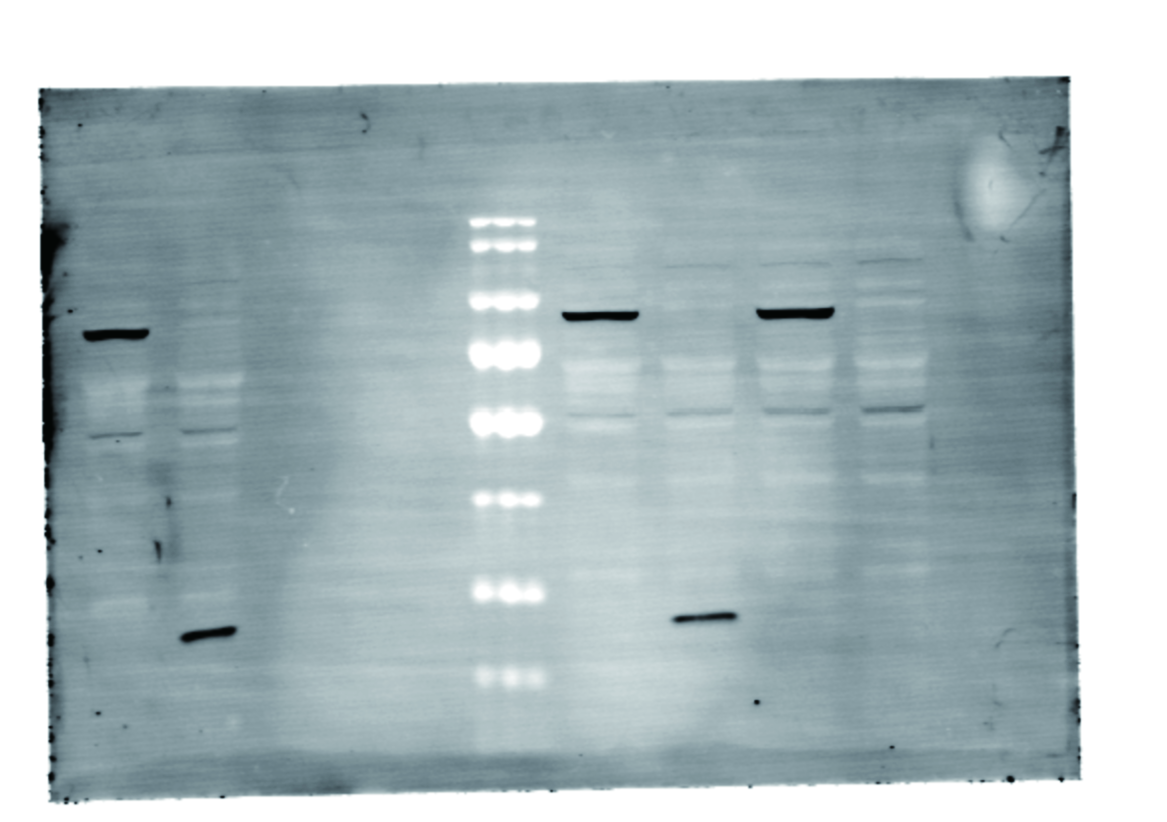

Supplement: Supplementary file 6 — Source data Fig. 3 [file 44318_2024_223_MOESM6_ESM.zip › figure 3/B/coIP nsf1.tif]

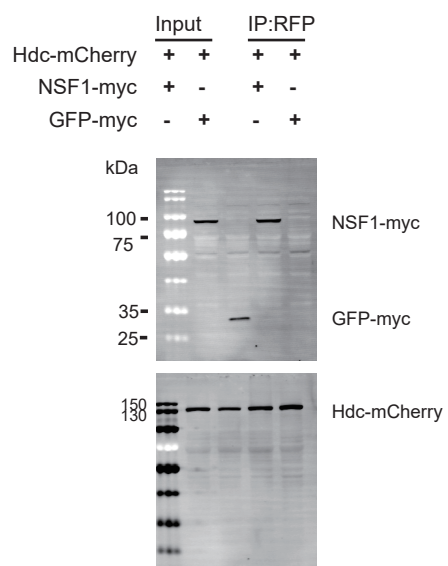

Supplement: Supplementary file 6 — Source data Fig. 3 [file 44318_2024_223_MOESM6_ESM.zip › figure 3/B/coIP.pdf]

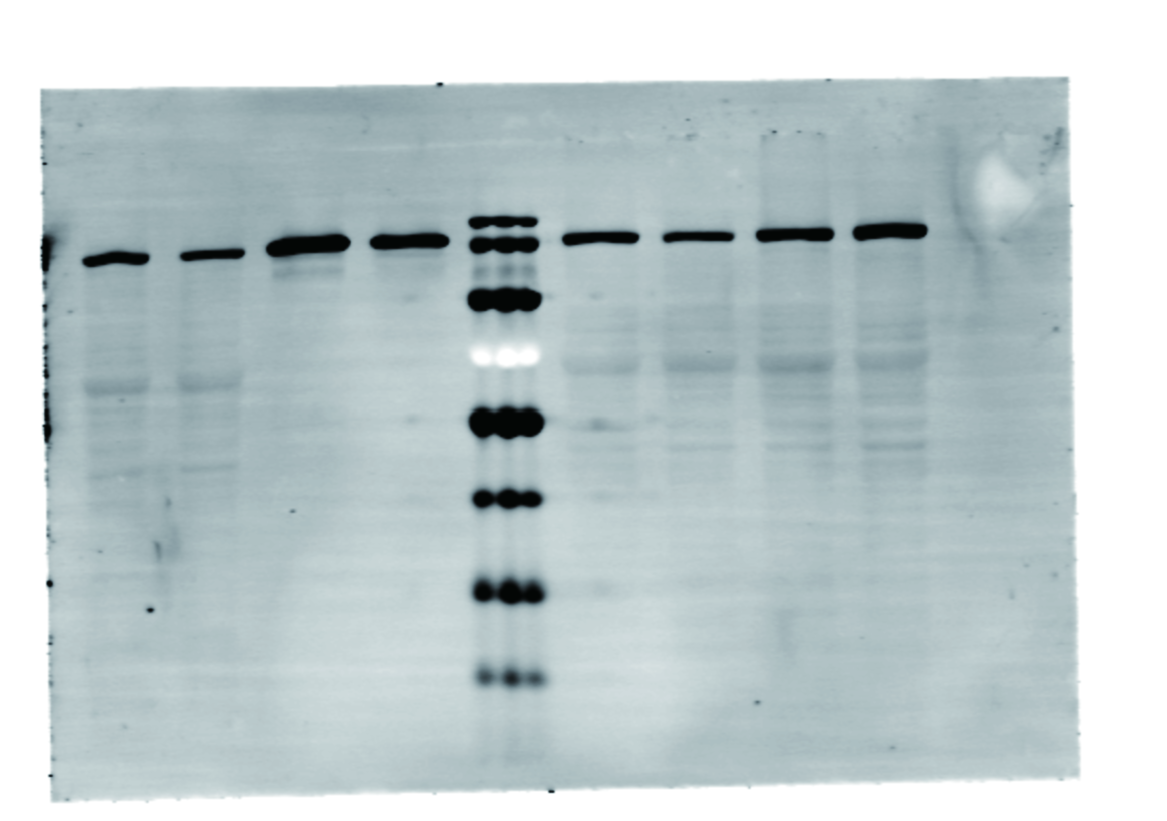

Supplement: Supplementary file 6 — Source data Fig. 3 [file 44318_2024_223_MOESM6_ESM.zip › figure 3/B/coip hdc.tif]

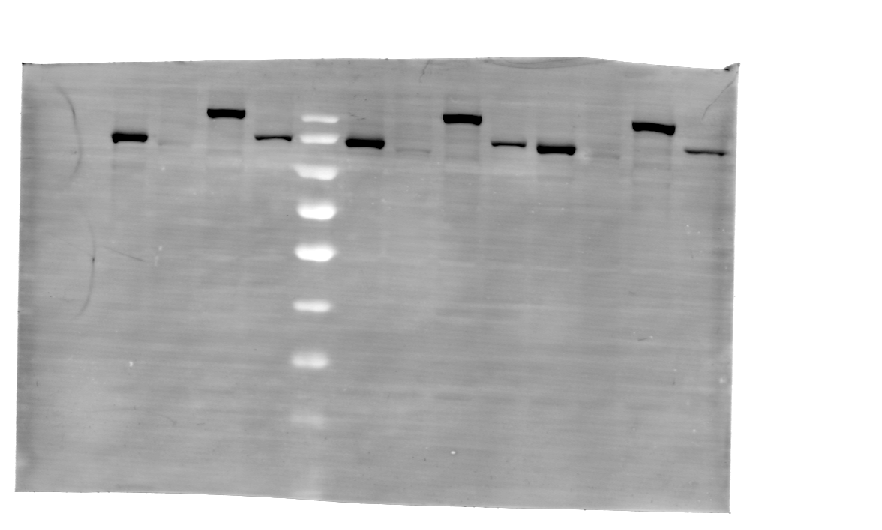

Supplement: Supplementary file 7 — Source data Fig. 4 [file 44318_2024_223_MOESM7_ESM.zip › figure 4/A/mcherry800.tif]

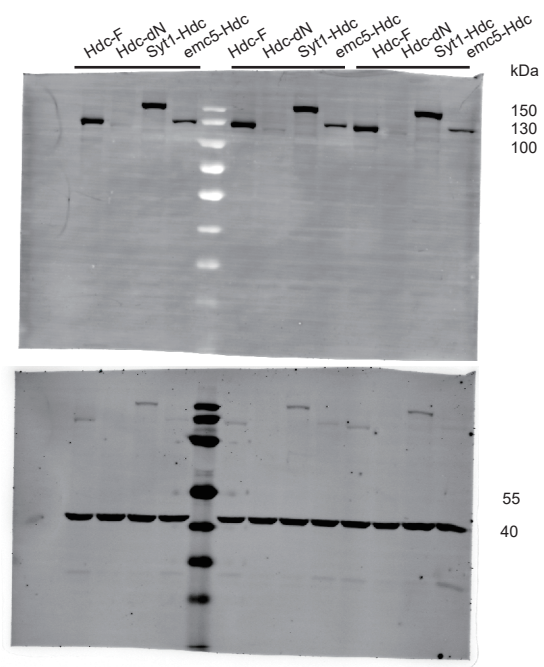

Supplement: Supplementary file 7 — Source data Fig. 4 [file 44318_2024_223_MOESM7_ESM.zip › figure 4/A/wb.pdf]

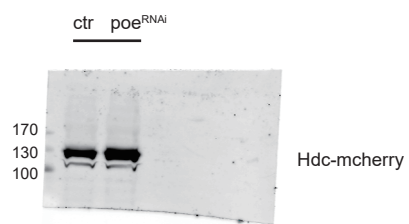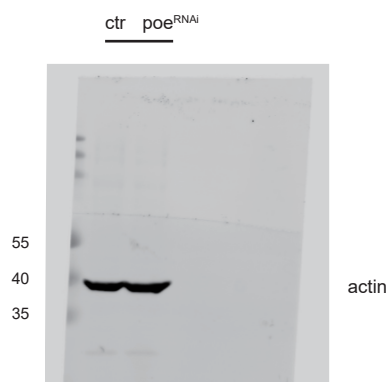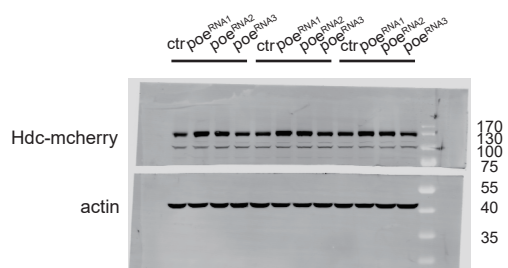

Supplement: Supplementary file 7 — Source data Fig. 4 [file 44318_2024_223_MOESM7_ESM.zip › figure 4/C/poeRNAi.pdf]

ctr a5 b1 atg7 atg8   ctr a5 b1 atg7 atg8

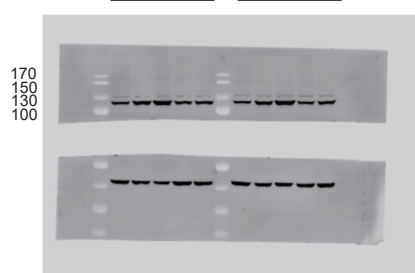

ctr a5 b1 atg7 atg8

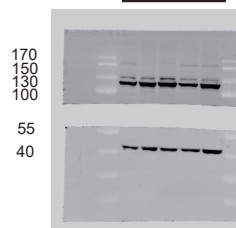

Supplement: Supplementary file 7 — Source data Fig. 4 [file 44318_2024_223_MOESM7_ESM.zip › figure 4/B/proteasome or autophage.pdf]

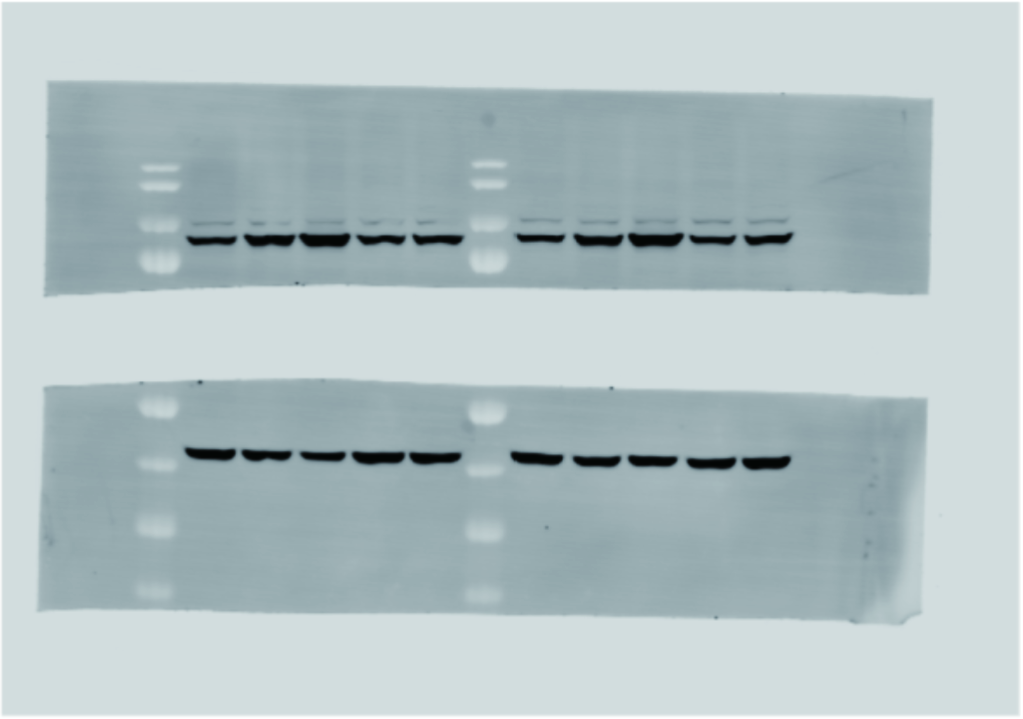

Supplement: Supplementary file 7 — Source data Fig. 4 [file 44318_2024_223_MOESM7_ESM.zip › figure 4/B/ctr a1 b5 atg7 atg8.tif]
