# Supplementary material for: Histamine synthesis and transport are coupled in axon terminals via a dual quality control system
Source: EMBO J. 2024 Sep 6;43(20):4. doi: 10.1038/s44318-024-00223-0 (PMC11480334; doi:10.1038/s44318-024-00223-0)
Supplement: Supplementary file 9 — Expanded View Figures [file 44318_2024_223_MOESM9_ESM.pdf]

Expanded View Figures

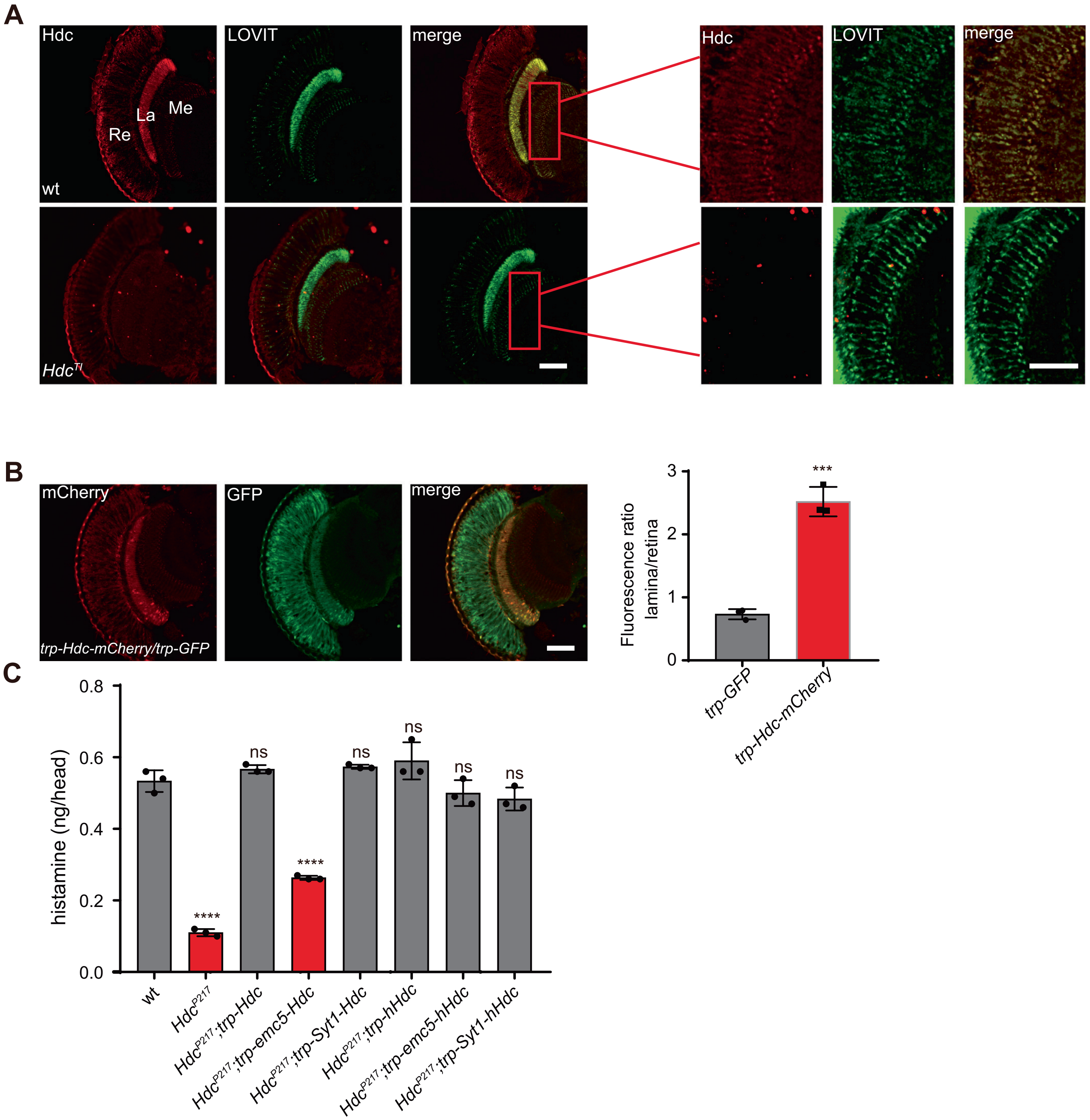

Figure EV1. Expression of Hdc in both soma and axon restores total histamine content.

(**A**) Head longitudinal sections of wild-type (wt) and Hdc null mutants (*Hdc*^{TI}) were labeled with Hdc and LOVIT antibodies. Sections of the distal medulla neuropil are shown on the right. Scale bars, 20 μm on the left and 50 μm on the right. La lamina, Me medulla, Re retina. (**B**) Head longitudinal sections from flies expressing both Hdc-mCherry and GFP in photoreceptor cells under the control of the *trp* promoter (*trp-Hdc-mCherry/trp-GFP*). Sections were labeled with antibodies against mCherry and GFP. *trp-GFP* vs *trp-Hdc-mCherry* ***$p = 0.0002$. Unpaired *t*-test, $n = 3$, mean ± sd. Scale bars, 20 μm. (**C**) Total histamine levels in heads of wild-type, Hdc mutant flies (*Hdc*^{P217}), and *Hdc*^{P217} flies expressing mCherry-tagged Hdc (*Hdc*^{P217}*;trp-Hdc*), EMC5-Hdc (*Hdc*^{P217}*;trp-emc5-Hdc*), Syt1-Hdc (*Hdc*^{P217}*;trp-Syt1-Hdc*), hHdc (*Hdc*^{P217}*;trp-hHdc*), EMC5-hHdc (*Hdc*^{P217}*;trp-emc5-hHdc*), or Syt1-hHdc (*Hdc*^{P217}*;trp-Syt1-hHdc*) driven by the *trp* promoter. Each sample contained 20 fly heads and the mean values from three samples were calculated. wt vs *Hdc*^{P217} ****$p < 0.0001$, wt vs *Hdc*^{P217}*;trp-Hdc* $p = 0.5571$, wt vs *Hdc*^{P217}*;trp-emc5-Hdc* ****$p < 0.0001$, wt vs *Hdc*^{P217}*;trp-Syt1-Hdc* $p = 0.3802$, wt vs *Hdc*^{P217}*;trp-hHdc* $p = 0.1151$, wt vs *Hdc*^{P217}*;trp-emc5-hHdc* $p = 0.5571$, wt vs *Hdc*^{P217}*;trp-Syt1-hHdc* $p = 0.1917$. Dunnett's one-way ANOVA, mean ± sd, ns not significant.

 © The Author(s)

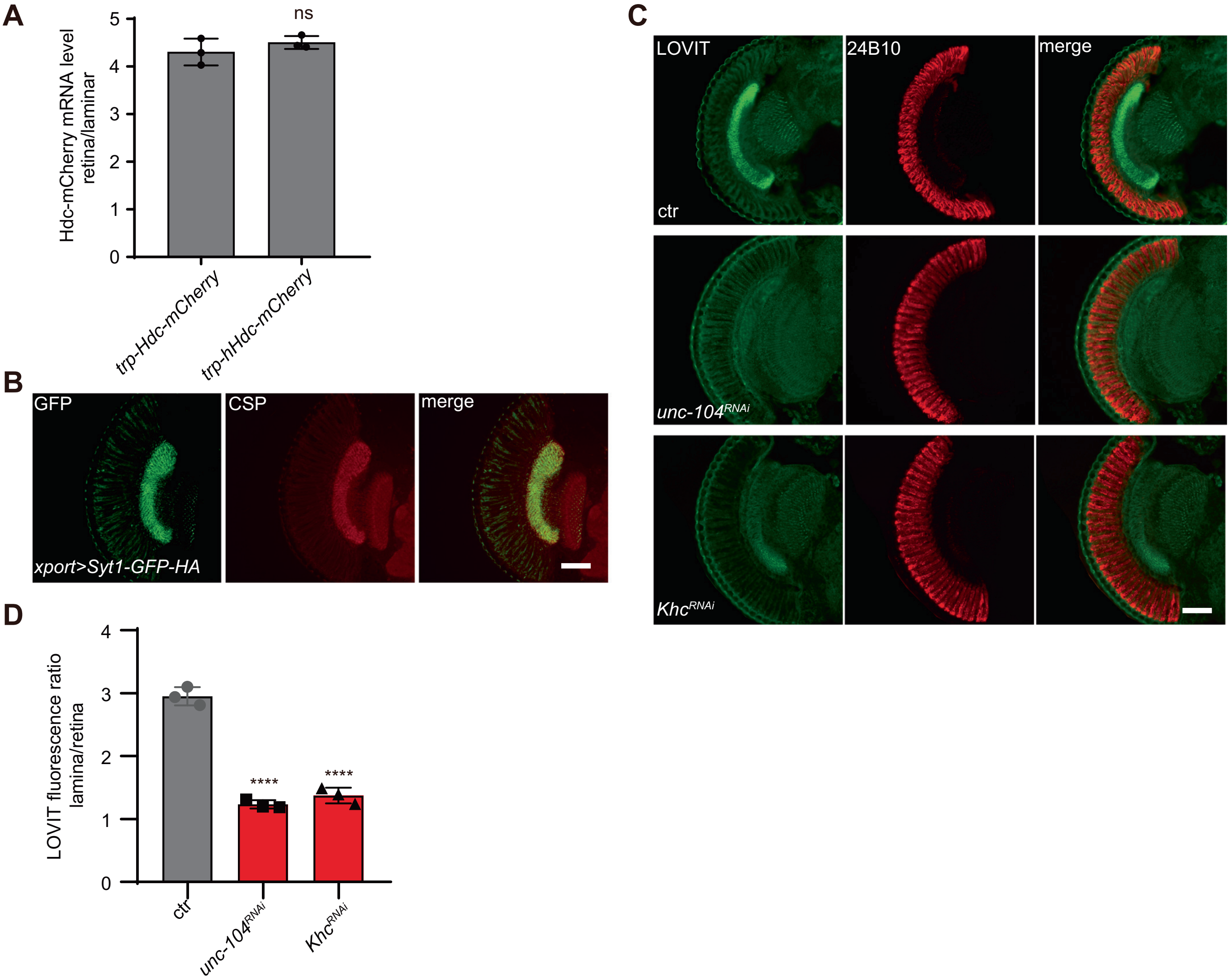

Figure EV2. Disruption of the kinesin motor reduces axonal synaptic vesicles.

(**A**) Hdc-mCherry mRNA level ratio between the lamina and retina of *trp-Hdc-mCherry* and *trp-hHdc-mCherry* flies. $p = 0.3287$. Unpaired *t*-test, $n = 3$, mean ± sd, ns not significant. (**B**) Immunostaining of the head section of *xport>Syt1-GFP-HA* flies (*xport-Gal4/UAS-Syt1-GFP-HA*) showed Syt1-GFP-HA colocalized with the synaptic vesicle marker, CSP. Scale bars, 20 μm. (**C**) Head cross-sections of control (*GMR>GFP*^{RNAi}, *GMR-Gal4/UAS-GFP*^{RNAi}), *GMR>unc-104*^{RNAi} (*GMR-Gal4/UAS-unc-104*^{RNAi}), and *GMR>Khc*^{RNAi} (*GMR-Gal4/UAS-Khc*^{RNAi}) were labeled with LOVIT antibodies (synaptic vesicles marker, green) and 24B10 (photoreceptor marker, red). Scale bars, 20 μm. (**D**) Quantification of LOVIT signals in lamina versus retina. Three fly head sections were used for quantification. ctr vs *unc-104*^{RNAi} ****$p < 0.0001$, ctr vs *Khc*^{RNAi} ****$p < 0.0001$. Dunnett's one-way ANOVA, mean ± sd.

© The Author(s)

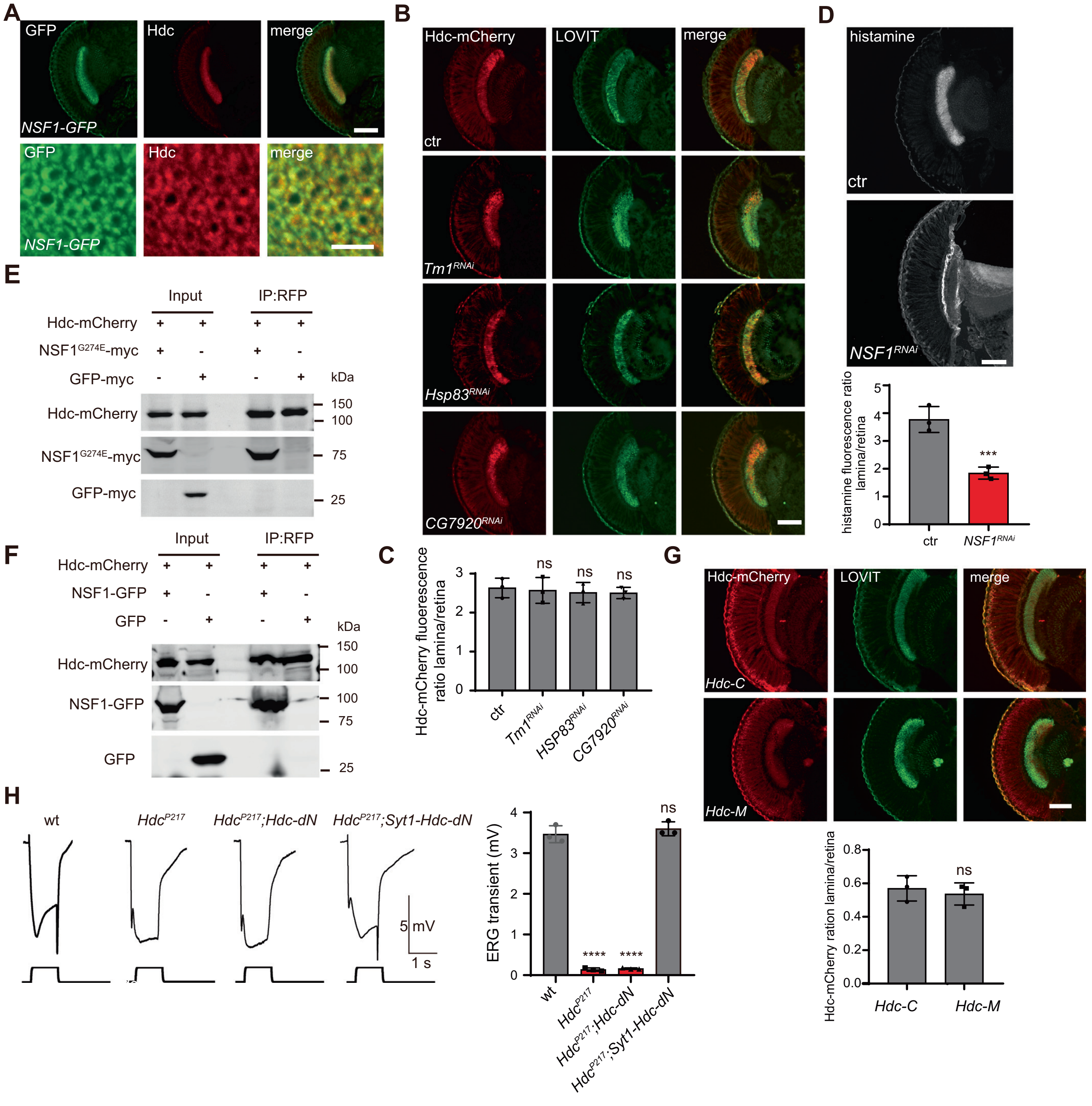

Figure EV3. Interaction between NSF1 and Hdc through its N-terminals is required for localization and function of Hdc at synaptic terminals.

(**A**) Head horizontal sections of flies expressing GFP-tagged NSF1 under a weak photoreceptor cell promoter (*culd-NSF1-GFP*) were labeled for GFP and Hdc. Cross-sections of the lamina are shown on the bottom. Scale bars, 20 µm at the top and 50 µm at the bottom. (**B**) Head sections of *trp-Hdc-mCherry* flies expressing GFP^{RNAi} (*GMR-Gal4/UAS-*GFP^{RNAi}), $Tm1^{RNAi}$ (*GMR-Gal4/UAS-*$Tm1^{RNAi}$), $Hsp83^{RNAi}$ (*GMR-Gal4/UAS-*$Hsp83^{RNAi}$), or $CG7920^{RNAi}$ (*GMR-Gal4/UAS-*$CG7920^{RNAi}$) were stained with mCherry antibodies. Scale bar, 20 µm. (**C**) Quantitation of Hdc-mCherry fluorescence ratio between lamina and retina. ctr vs $Tm1^{RNAi}$ $p = 0.9871$, ctr vs $Hsp83^{RNAi}$ $p = 0.8907$, ctr vs $CG7920^{RNAi}$ $p = 0.8675$. Dunnett's one-way ANOVA, $n = 3$, mean ± sd, ns not significant. (**D**) Horizontal sections of wild-type (wt) and flies expressing $NSF1^{RNAi1}$ ($NSF1^{RNAi}$) were labeled using antibodies against histamine. Scale bars, 20 µm. Quantification of fluorescence intensity ratios of histamine between the lamina and retina was shown on the right. ctr vs $NSF1^{RNAi}$ ***$p = 0.0005$. Unpaired *t*-test, $n = 3$, mean ± sd. (**E**) Interaction of Hdc and NSF1 is independent of the ATPase activity of NSF1. Hdc-mCherry was co-expressed with $NSF1^{G274E}$-Myc or GFP-Myc in S2 cells. Cell lysates were immunoprecipitated with anti-mCherry beads, and blotted against either mCherry or Myc. (**F**) Hdc interacts with NSF1 in vivo. Cell lysates from dissected heads of *trp-Hdc-mCherry/culd-NSF1-GFP* flies or *trp-Hdc-mCherry/trp-GFP* flies were immunoprecipitated with anti-mCherry beads, and blotted against mCherry and GFP. (**G**) Immunostaining of head sections of flies expressing mCherry-tagged truncated Hdc-M (*trp-Hdc-M-mCherry*) and Hdc-C (*trp-Hdc-C-mCherry*) showed a reduction of mCherry signals in lamina. Hdc-C vs Hdc-M $p = 0.5970$. Unpaired *t*-test, $n = 3$, mean ± sd, ns not significant. (**H**) ERG recorded from Hdc^{P217} flies expressing Hdc-dN (Hdc^{P217}*;trp-Hdc-dN*) and Syt1-Hdc-dN (Hdc^{P217}*;trp-Syt1-Hdc-dN*). OFF transients were quantified based on data from three flies. wt vs Hdc^{P217} ****$p < 0.0001$, wt vs Hdc^{P217}*;trp-Hdc-dN* ****$p < 0.0001$, wt vs Hdc^{P217}*;trp-Syt1-Hdc-dN* $p = 0.5272$. Dunnett's one-way ANOVA, mean ± sd, ns not significant.

 © The Author(s)

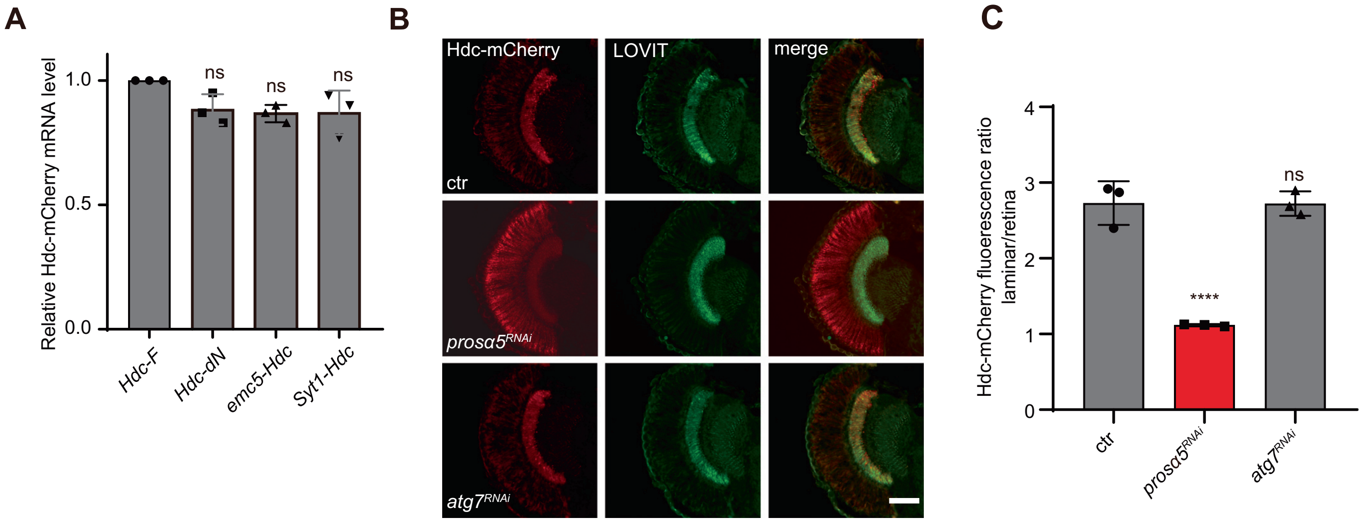

Figure EV4. Disruption of the proteasome but not lysosomes increases Hdc levels in the retina.

(**A**) QPCR comparison of mRNA levels of truncated Hdc. Total RNA was extracted from heads of Hdc-F (*trp-Hdc-mCherry*), Hdc-dN (*trp-Hdc-dN-mCherry*), emc5-Hdc (*trp-emc5-Hdc-mCherry*), and Syt1-Hdc (*trp-Syt1-Hdc-mCherry*) flies. A primer pair against *mCherry* sequences was used for QPCR, and results were normalized to *rp49*. Hdc-F vs Hdc-dN $p = 0.0854$, Hdc-F vs emc5-Hdc $p = 0.0504$, Hdc-F vs Syt1-Hdc $p = 0.0557$. Dunnett's one-way ANOVA, $n = 3$, mean ± sd, ns not significant. (**B**) Head horizontal sections of *trp-Hdc-mCherry* flies expressing GFP^{RNAi} ($GMR>GFP^{RNAi}$, *trp-Hdc-mCherry GMR-Gal4/UAS-*GFP^{RNAi}), $prosα5^{RNAi}$ ($GMR>prosα5^{RNAi}$, *trp-Hdc-mCherry GMR-Gal4/UAS-*$prosα5^{RNAi}$), and $atg7^{RNAi}$ ($GMR>atg7^{RNAi}$, *trp-Hdc-mCherry GMR-Gal4/UAS-*$atg7^{RNAi}$) were labeled with mCherry (red) and LOVIT (green) antibodies. Scale bars, 20 µm. (**C**) Quantification of Hdc-mCherry fluorescence ratio between lamina and retina. Three fly head sections were used for quantification. ctr vs $prosα5^{RNAi}$ ****$p < 0.0001$, ctr vs $atg7^{RNAi}$ $p = 0.9986$. Dunnett's one-way ANOVA, mean ± sd, ns not significant.

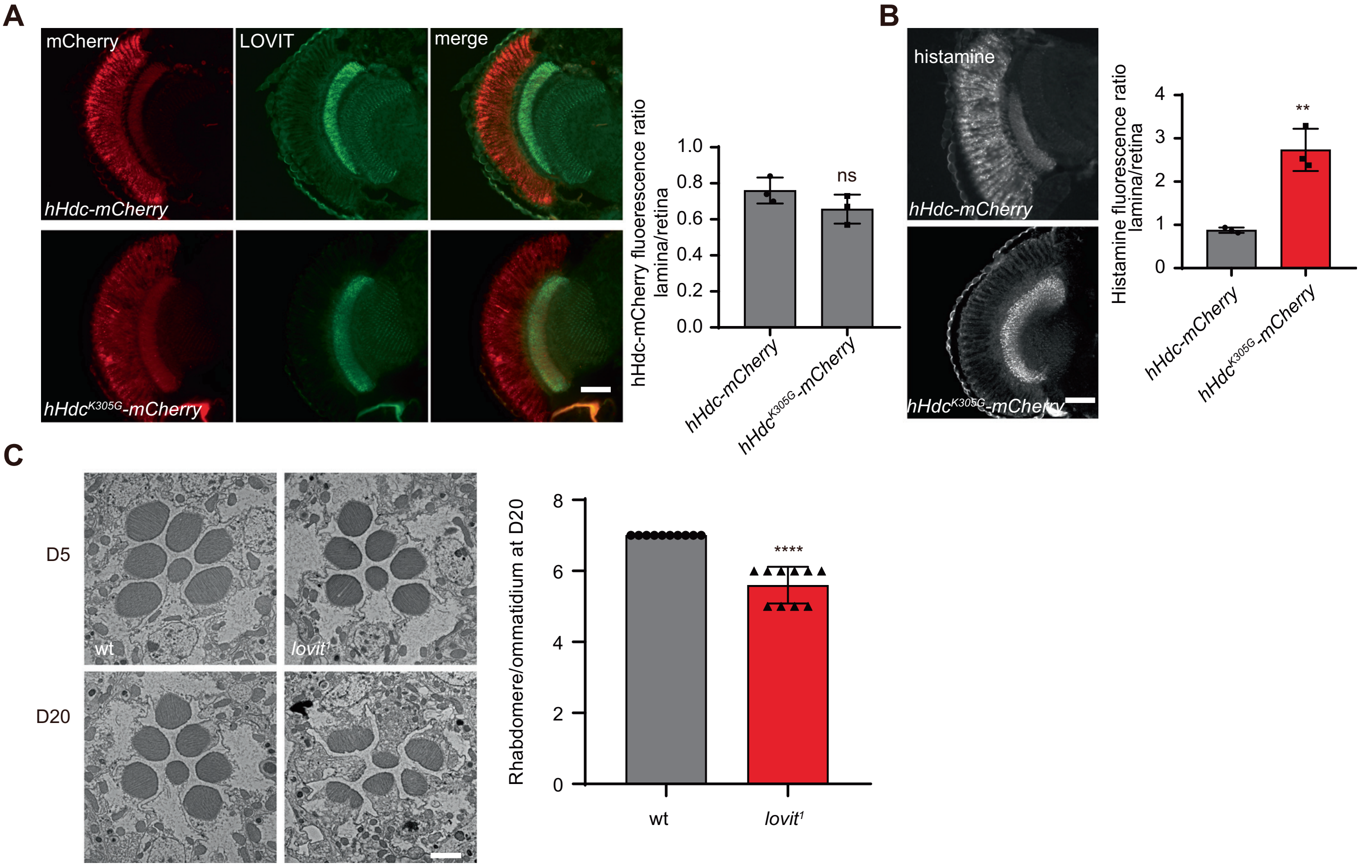

Figure EV5. The Hdc^{K305G} mutant cannot synthesize histamine.

(**A**) Immunostaining of mCherry in *trp-hHdc-mCherry* and *trp-hHdc^{K305G}-mCherry* flies revealed no changes in the distribution and levels of ectopic hHdc proteins. *hHdc-mCherry* vs *hHdc^{K305G}-mCherry* $p = 0.1738$. Unpaired *t*-test, $n = 3$, mean ± sd. (**B**) Histamine was immunolabeled in horizontal sections of heads of *trp-hHdc-mCherry* and *trp-hHdc^{K305G}-mCherry* flies. Quantification of histamine fluorescence intensity ratios between the entire lamina and retina were based on three sections. *hHdc-mCherry* vs *hHdc^{K305G}-mCherry* $^{**}p = 0.0028$. Unpaired *t*-test, mean ± sd. (**C**) TEM images from wildtype and *lovit^1* flies that were 1 (upper) or 20 days old (bottom). Scale bar, 2 µm. Ten sections were used for quantification. wt vs *lovit^1* $^{****}p < 0.0001$. Unpaired *t*-test, mean ± sd.

 © The Author(s)
